# The NTCP p.Ser267Phe Variant Is Associated With a Faster Anti-HBV Effect on First-Line Nucleos(t)ide Analog Treatment

**DOI:** 10.3389/fphar.2021.616858

**Published:** 2021-02-24

**Authors:** Lina Wu, Wenxiong Xu, Xuejun Li, Ying Liu, Lu Wang, Shu Zhu, Fangji Yang, Chan Xie, Liang Peng

**Affiliations:** ^1^ Department of Infectious Diseases, Third Affiliated Hospital of Sun Yat-sen University, Guangzhou, China; ^2^ Guangdong Province Key Laboratory of Liver Disease Research, The Third Affiliated Hospital of Sun Yat-sen University, Guangzhou, China

**Keywords:** NTCP, nucleos(t)ide analogues, chronic hepatitis B (CHB), single nuceotide polymorphism, HBV DNA

## Abstract

Sodium taurocholate cotransporting polypeptide (NTCP) acts as a cellular receptor for the hepatitis B virus infection of host hepatocytes. Previously, many studies confirmed that the NTCP p.Ser267Phe variant was a protective factor against HBV-related disease progression. We therefore designed this study to investigate whether the NTCP p.Ser267Phe variant exerts an additive anti-HBV effect in chronic hepatitis B (CHB) patients on mainstream NAs treatment. After propensity score matching (PSM), a total of 136 CHB patients were included, among whom 68 were heterozygous carriers and 68 were wild-type controls. Proportions of primary nonresponse, partial virological response, virological breakthrough and hepatitis B reactivation and the HBV DNA clearance rate at each time point were compared using the chi-square test. Kaplan-Meier analysis and matched t-tests were also performed to estimate the speed of viral clearance and serum HBV DNA reduction, respectively. The proportion of primary nonresponse was significantly lower in heterozygous carriers than in wild-type controls (*p* < 0.001), especially in patients using entecavir (*p* = 0.013). Specifically, heterozygous carriers achieved HBV DNA clearance faster than wild-type controls (log-rank *p* = 0.0198). HBV DNA levels were reduced more in heterozygous carriers after 12 weeks (*p* < 0.001) and 24 weeks (*p* = 0.006) of treatment, especially among patients using ETV. Here, our study demonstrated that heterozygous mutations in rs2296651 enhanced the antiviral response of first-line NAs and helped to explore the possibility of combining NAs and NTCP blockers for a better anti-HBV effect.

## Introduction

Hepatitis B virus (HBV) infection is a public health challenge that affects more than 250 million people worldwide (WHO, 2017). Oral nucleos(t)ide analog (NA) therapy is currently the mainstream form of anti-HBV treatment. Viral suppression with NAs therapy directly affects the prognosis of CHB patients. Currently, the recommended first-line choice includes entecavir (ETV), tenofovir disoproxil fumarate (TDF) and tenofovir alafenamide (TAF) (Seto et al., 2018). All patients included in this study received either the nucleoside analog ETV or nucleotide analog TDF (Chen and Liaw, 2016). Biochemically, these triphosphorylated NAs compete with endogenous deoxynucleoside triphosphates (dNTPs) for incorporation into the viral DNA chain and terminate its elongation immediately (Fung et al., 2011) or at 2 or 3 nt downstream of incorporation (ETV) (Langley et al., 2007). Because viral DNA polymerase lacks a proofreading function (3′ exonuclease activity), incorporated NAs cannot be removed, and thus, viral DNA synthesis is irreversibly terminated. Such terminated viral DNA results in generations of HBV DNA in intracellular nucleocapsids and secreted virion-like particles spreading into liver cells that are not infected through sodium taurocholate cotransporting polypeptide (NTCP).

NTCP, encoded by SLC10A1, is a sodium–bile acid cotransporter that mediates the reuptake of circulating bile acids from the portal blood into the liver. The process through which HBV enters human hepatocytes begins with the binding of HBV preS1-polypeptide and its receptor sodium taurocholate cotransporting polypeptide (NTCP) expressed by the host (Yan et al., 2014). The newly developed NTCP blocker Myrcludex B is a chemically synthesized lipopeptide that comprises parts of the pre-S1 domain of the hepatitis B virus (HBV) large surface protein (Blank et al., 2018) and is currently in phase 2b and phase 3 clinical trials for the treatment of HBV and HDV infections, respectively. Directly targeting NTCP, Myrcludex B reduced the HBV load by preventing HBV from entering hepatocytes (Zhao et al., 2019).

Along with for NTCP blockers, previous studies demonstrated that specific single-nucleotide polymorphisms (SNPs), especially NTCP S267F, altered the physiological function of NTCP, including bile salt homoeostasis, HBV entry, and clinical outcomes of HBV infection (Yan et al., 2014; Peng et al., 2015; Liu et al., 2017; Yang et al., 2019). Most SLC10A1 SNPs have distributions related to ethnicity, and the nonsynonymous mutation that encodes the p.Ser267Phe variant (S267F, c.800 G>A, rs2296651) (minor allele frequency from the 1,000 Genomes Browser is 0.0144) is specific to Asian patient populations (Pan et al., 2011). The p.Ser267Phe variant of NTCP abolishes or reduces HBV infection *in vivo* and *in vitro* (Yan et al., 2014; Peng et al., 2015). Our latest study found that the NTCP p.Ser267Phe variant is inversely associated with HBV-related disease progression (Yang et al., 2019), in accordance with several other studies (Wang et al., 2017),we therefore designed this study to investigate whether the NTCP p.Ser267Phe variant exerts an additive anti-HBV effect in chronic hepatitis B (CHB) patients on mainstream NAs treatment.

Since the p.Ser267Phe variant of NTCP can prevent HBV entry or reduce the amount of HBV entering hepatocytes *in vivo* and *in vitro* and act as an entry inhibitor to a certain extent, while NAs inhibit HBV replication, perhaps the NTCP p.Ser267Phe variant exerts a protective effect on CHB patients partly by enhancing the antiviral effect in combination with NAs. This study aimed to investigate whether the rs2296651 mutation in NTCP leads to a better antiviral effect than wild-type controls in CHB patients on first-line NAs therapy and to explore the possibility of combining first-line NAs and NTCP blockers for a better anti-HBV effect.

### Patients and Methods

#### Inclusion/Exclusion Criteria and Cohort Building

From May 2008 to December 2019, a total of 3,464 individuals with chronic HBV infection, of whom 303 were heterozygous (rs2296651 GA) and 6 were homozygous (rs2296651 AA), from the Third Affiliated Hospital of Sun Yat-Sen University in Guangzhou, Guangdong Province, China, were enrolled in our previous and ongoing studies related to the NTCP p.Ser267Phe variant. All cases were diagnosed with CHB based on seropositivity of B surface antigen (HBsAg) for over 6 months in accordance with the Chinese Guidelines for the Prevention and Treatment of CHB (Chinese Society Of Hepatology, 2019). Patients who were pregnant; coinfected with hepatitis A, C, D, E, or human immunodeficiency virus; and suffering from autoimmune diseases, alcohol or drug-induced hepatitis or fatty liver disease were excluded. Patients were first placed into 3 diagnosis groups: cirrhosis, hepatocyte carcinoma, and liver failure (the definitions of these diagnoses can be found in our previous article (Yang et al., 2019) or seen in Supplementary Table S2). The remaining patients for whom the presence of cirrhosis, hepatocyte carcinoma, and liver failure had been excluded were classified as CHB.

**FIGURE 1:**
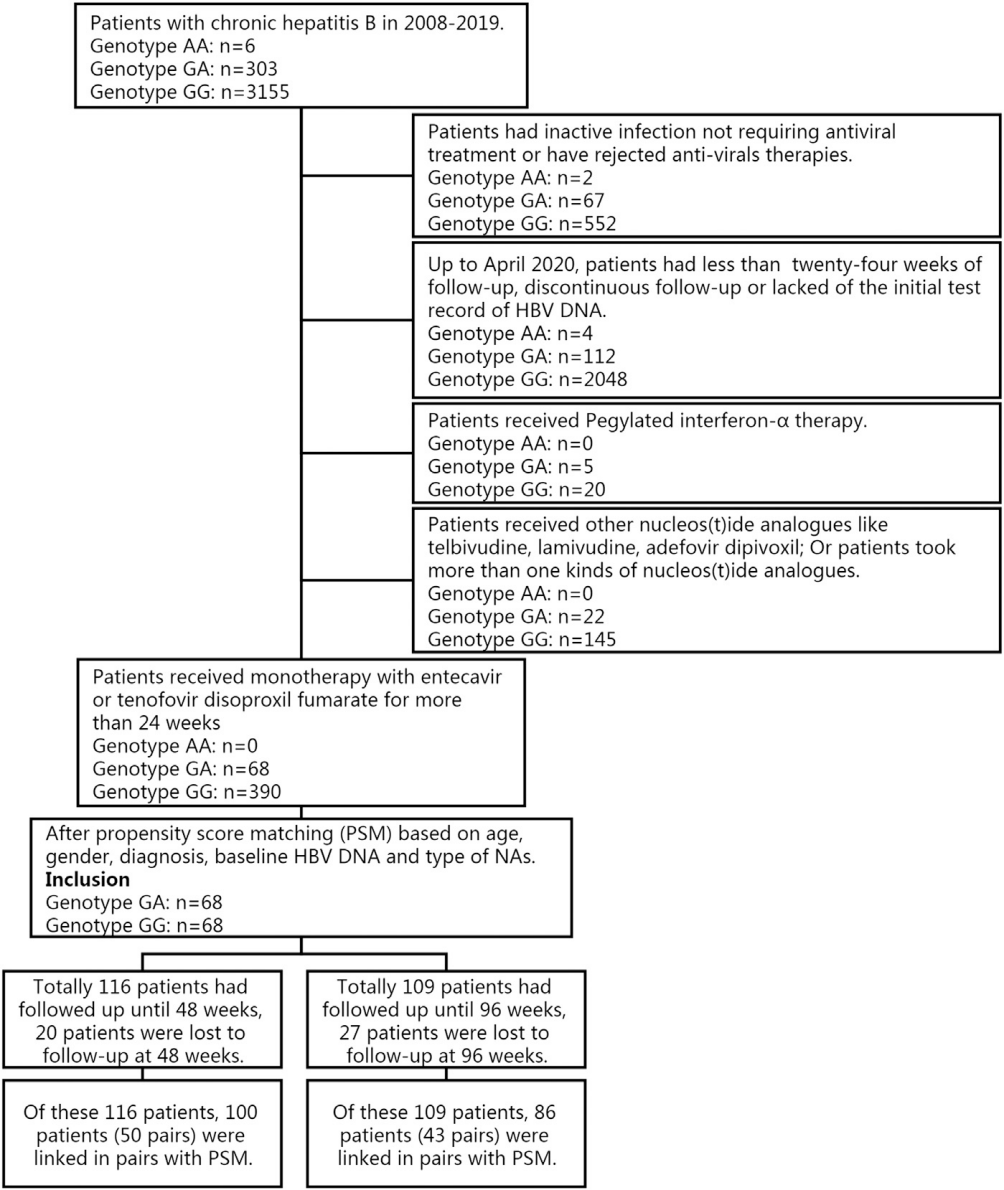
Flow chart of subjects enrollment for whose data were available.

CHB patients continuously using monotherapy of the first-line NAs entecavir or tenofovir disoproxil fumarate for at least 24 weeks were selected for propensity score matching (PSM) with IBM SPSS Statistics 25 software (IBM SPSS Statistics, IBM, Chicago, IL, United States) to control for confounding factors, such as age, sex, diagnosis, baseline HBV DNA and type of NAs (see Figure 1).

### Genotyping and Virological Response Evaluation

Rs2296651 genotyping was performed using a SNaPshot multiplex assay or Sanger sequencing (primer information is provided in Supplementary Table S1). SNPs were visualized using GeneMapper 4.1 (Applied Biosystems, Foster City, CA, USA). We assigned 4 outcomes, primary nonresponse (defined by a less than one log10 decrease of serum HBV DNA after 3 months or less than two log10 decrease of serum HBV DNA after 6 months of therapy) (Lampertico et al., 2017), partial virological response (defined as a decrease in HBV DNA of more than 2 log10 IU/ml but detectable HBV DNA after at least 6 months of therapy in compliant patients) (Hou and Lai, 2015), virological breakthrough (defined as a confirmed increase in HBV DNA level of more than 1 log10 IU/ml compared to the lowest HBV DNA level on-therapy) (Lampertico et al., 2017) and hepatitis reactivation (defined as a confirmed > 2 log10 IU/ml increasement of HBV DNA level in patients with previously stable undetected HBV DNA level on-therapy) (Hou and Lai, 2015), to evaluate the virological response (Hou and Lai, 2015; Lampertico et al., 2017). The HBV DNA load was determined using a Cobas HBV Amplicor Monitor assay (Roche Molecular Diagnostics, Branchburg, US). The lower and upper limits of detection were 20 IU/ml and 7 log10 IU/ml, respectively.

The baseline HBV DNA level was recorded before the beginning of NAs therapy. The time of HBV DNA clearance and HBV DNA level reduction compared to the baseline HBV DNA load at each time point for wild-type control and heterozygous cases were also documented for further analysis.

### Data Analysis

Data were analyzed with IBM SPSS Statistics 25 software (IBM SPSS Statistics, IBM, Chicago, IL, USA). For numeric variables, matched-pairs two-sample t-tests were used to compare normally distributed variables. Categorical variables were analyzed using the χ2 test or Fisher’s exact test. Kaplan-Meier curves were plotted to assess the cumulative rate of HBV clearance at each time point, and the difference between the groups was assessed by a log-rank test. All statistical tests were two-tailed, and a *p*-value less than 0.05 was defined as statistically significant.

Through TDF and ETV both exert anti-HBV effects via the replication process, they do not share the same mechanism (Fung et al., 2011). Therefore, we also conducted subgroup analysis for each nucleos(t)ide analog.

## Results

### Population Characteristics and Baseline

After propensity score matching, our study included 68 heterozygous carriers (rs2296651 GA) and 68 wild-type controls (rs2296651 GG) (see Figure 1). The demographic and clinical characteristics of the recruited population are summarized in Table 1. Participants had a mean age of 44.32 years and included 112 males and 25 females. All patients received first-line NAs (ETV and TDF), mostly ETV (80.88%), in this study. The baseline HBV DNA level was 5.94 log10 IU/mL, and the baseline HBsAg level was 5114.59 IU/ml. The between-group demographic and clinical characteristics were similar; therefore, their subsequent antiviral effects on HBV were comparable.

**TABLE 1:** Compared characteristics of wild-type control and heterozygote cases.

	All subjects(*N* = 136)	GG (*N* = 68)	GA (*N* = 68)	p#	Odds ratio (95%CI)
**Patients characteristic**					
Male (%)	112/136 (82.35)	55/68 (80.88)	57/68 (83.82)	0.822	-
Age, years	44.32 ± 11.18	44.56 ± 11.75	44.08 ± 10.66	0.428	-
Patients using ETV (%)	110/136 (80.88)	20/55 (36.36)	20/55 (36.36)	1.000	-
♣**Baseline**					
HBV DNA, log10 IU/mL	5.94 ± 1.69	5.79 ± 1.76	6.09 ± 1.62	0.220	-
HBsAg, IU/ml	5114.59 ± 9383.31	5373.11 ± 9709.35	6140.06 ± 11503.98	0.753	-

#*p* - value between GG and GA group. Significant p-value in bold (threshold p < 0.05)

♣The latest laboratory test results before initiation of NAs treatment was defined as baseline

### Association of the NTCP p.Ser267Phe Variant and viral Responses to First-Line NAs

**FIGURE 2:**
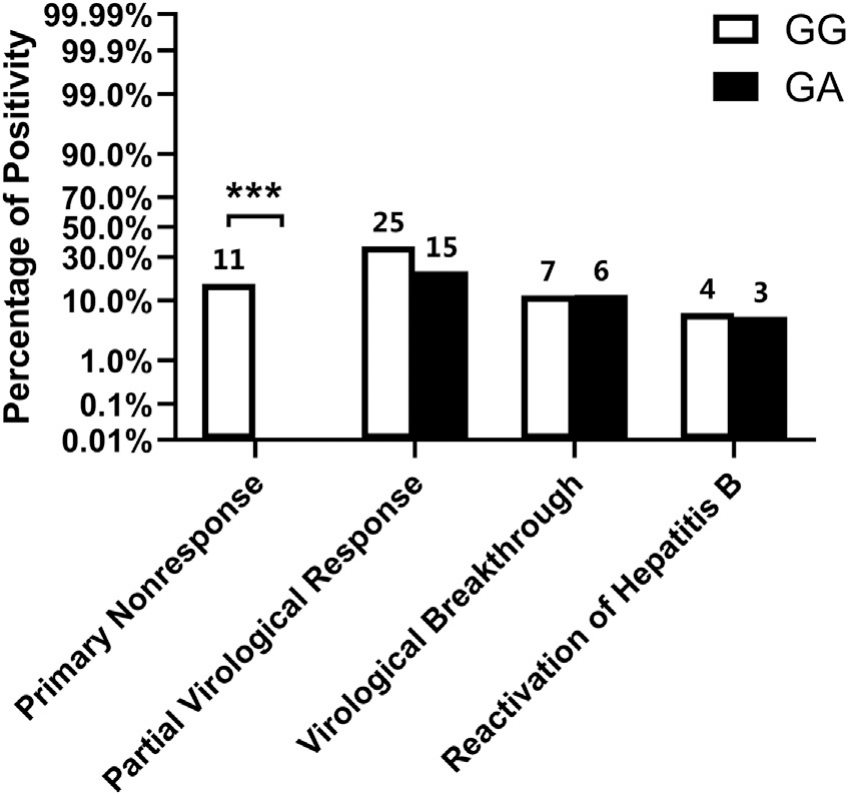
Virological outcome of patients using First-line NAs. Numbers above the bar indicate the specific frequency (ie., raw number of patients who were primary nonresponse in wild type controls).*p < 0.05, **p < 0.01, ***p < 0.001.

To investigate whether the NTCP p.Ser267Phe variant is related to the viral response, four antiviral outcomes were evaluated in heterozygous carriers and wild-type controls (see Table 2 and Figure 2). According to statistical analysis, no heterozygous carrier was found to be a primary nonresponder (OR, 0.84; 95% CI, 0.76–0.93; *p* < 0.001), and the rate of primary nonresponse in wild-type controls was 16.18%. However, though the between-group differences were not significant (*p* > 0.05), heterozygous carriers had fewer instances of partial virological response and hepatitis B reactivation than wild-type controls.

**TABLE 2:** Virological outcome of patients using First-line NAs in propensity matching cohort

	All subjects	GG	GA	p#	Odds ratio (95%CI)
**Primary nonresponse (%)**	11/136 (8.09)	11/68 (16.18)	0/68 (0)	**< 0.001**	**0.84 (0.76, 0.93)**
ETV (%)	7/110 (6.36)	7/55 (12.73)	0/55 (0)	**0.013**	**0.87 (0.79, 0.97)**
TDF (%)	4/26 (15.38)	4/13 (30.77)	0/13 (0)	0.096	0.69 (0.48, 1.00)
**Partial virological response (%)**	40/136 (29.41)	25/68 (36.76)	15/68 (22.06)	0.090	0.49 (0.23, 1.04)
ETV (%)	30/110 (27.27)	20/55 (36.36)	10/55 (18.18)	0.053	0.39 (0.16, 0.94)
TDF (%)	10/26 (38.46)	5/13 (38.46)	5/13 (38.46)	1.000	1.00 (0.21, 4.86)
**Virological breakthrough (%)**	13/111 (11.71)	7/60 (11.67)	6/51 (11.76)	1.000	1.01 (0.32, 3.22)
ETV (%)	10/92 (10.87)	6/50 (12)	4/42 (9.52)	0.750	0.77 (0.20, 2.94)
TDF (%)	3/19 (15.79)	1/10 (10)	2/9 (22.22)	0.582	2.57 (0.19, 34.47)
**Reactivation of hepatitis B (%)**	7/111 (6.31)	4/60 (6.67)	3/51 (5.88)	1.000	0.88 (0.19, 4.11)
ETV (%)	6/92 (6.52)	4/50 (8)	2/42 (4.76)	0.684	0.58 (0.10, 3.31)
TDF (%)	1/19 (5.26)	0/10 (0)	1/9 (11.11)	0.474	1.13 (0.89, 1.42)

#*p* - value between GG and GA group. Significant p-value in bold (threshold p < 0.05).

**FIGURE 3:**
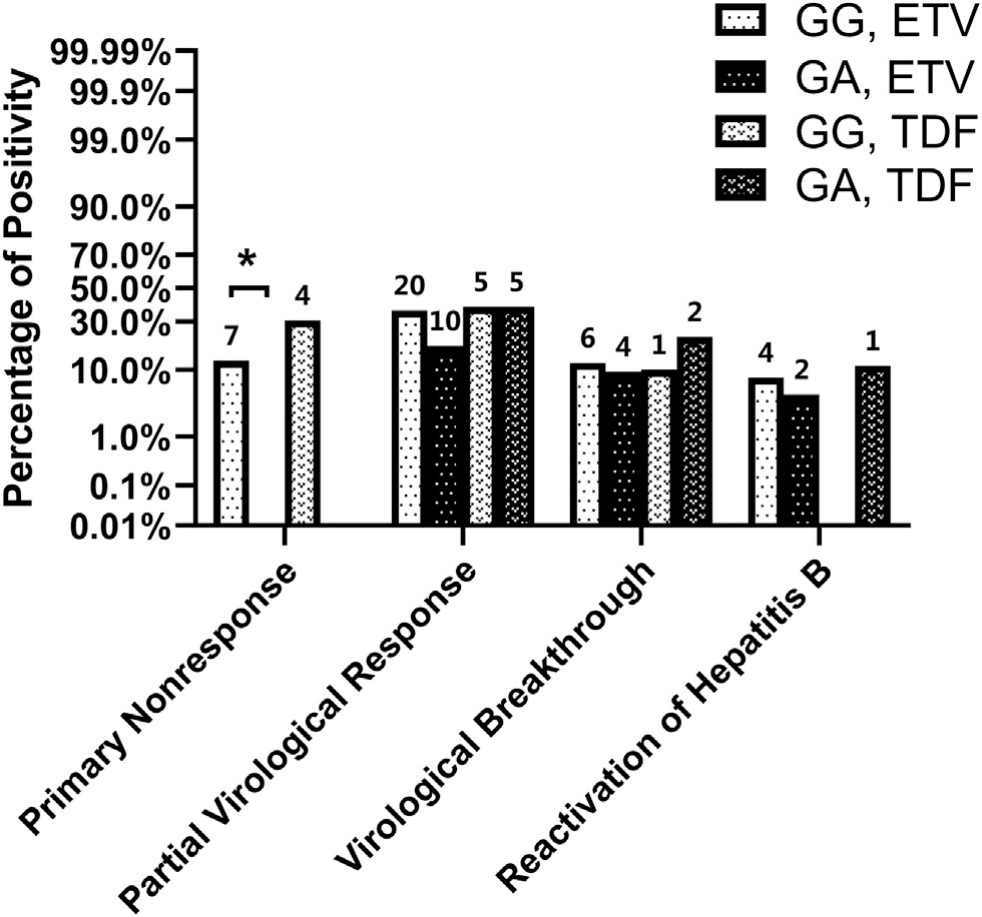
Virological outcome of patients using ETV or TDF.

For subgroup analysis (see Table 2 and Figure 3), even though the proportions of primary nonresponse, partial virological response, and hepatitis B reactivation were lower in heterozygous carriers than in wild-type controls among patients taking ETV, only the percentage of primary nonresponse was found to be significantly lower (OR, 0.87; 95% CI, 0.79–0.97; *p* = 0.013). Conversely, among patients taking TDF, though the differences were not significant, patients carrying the NTCP p.Ser267Phe variant showed more occurrences of events related to HBV relapse (virological breakthrough and hepatitis B reactivation) than the wild-type patients.

Thus, patients with the NTCP p.Ser267Phe variant are less likely to be primary nonresponders to first-line NAs therapy, especially those using ETV.

Association between the NTCP p.Ser267Phe variant and HBV DNA clearance in CHB patients on first-line NAs treatment

To further investigate associations between the NTCP p.Ser267Phe variant and the difference in HBV DNA seronegativity, the cumulative rate of HBV clearance at 12, 24, 48, and 96 weeks were determined and compared between groups (see Table 3). According to statistical analysis, 67.65% of mutation carriers had undetectable HBV DNA levels at 12 weeks, which was significantly higher than the 39.71% of wild-type controls (OR, 1.86; 95% CI, 1.26–2.76; *p* = 0.002). However, even though the cumulative proportion of HBV clearance was higher in heterozygous carriers than in wild-type controls at 24, 48, and 96 weeks, the difference was not significant (*p* ≥ 0.05).

**TABLE 3:** Percentage of ‡HBV DNA Clearance at each time point.

	All subjects	GG	GA	p#	Odds ratio (95%CI)
**12-weeks (%)**	73/136 (53.68)	27/68 (39.71)	46/68 (67.65)	**0.002**	**1.86 (1.26, 2.76)**
ETV (%)	60/110 (54.55)	20/55 (36.36)	40/55 (72.73)	**<0.001**	**2.33 (1.45, 3.75)**
TDF (%)	13/26 (50)	7/13 (53.85)	6/13 (46.15)	1.000	0.74 (0.16, 3.43)
**24-weeks (%)**	96/136 (70.59)	43/68 (63.24)	53/68 (77.94)	0.090	2.05 (0.97, 4.38)
ETV (%)	80/110 (72.73)	35/55 (63.64)	45/55 (81.82)	0.053	2.57 (1.07, 6.19)
TDF (%)	16/26 (61.54)	8/13 (61.54)	8/13 (61.54)	1.000	1.00 (0.21, 4.86)
**48-weeks (%)**	112/136 (82.35)	53/68 (77.94)	59/68 (86.76)	0.261	1.86 (0.75, 4.59)
ETV (%)	91/110 (82.73)	43/55 (78.18)	48/55 (87.27)	0.313	1.91 (0.69, 5.30)
TDF (%)	21/26 (80.77)	10/13 (76.92)	11/13 (84.62)	1.000	1.65 (0.23, 12.00)
**96-weeks (%)**	129/136 (94.85)	63/68 (92.65)	66/68 (97.06)	0.441	1.68 (0.52, 5.42)
ETV (%)	104/110 (94.55)	51/55 (92.73)	53/55 (96.36)	0.742	1.56 (0.42, 5.87)
TDF (%)	25/26 (96.15)	12/13 (92.31)	13/13 (100)	1.000	2.18 (0.17, 27.56)

#*p* - value between GG and GA group. Significant p-value in bold (threshold p < 0.05).

^‡^HBV DNA clearance defined as an undetectable HBV DNA load (<20 IU/ml) in peripheral blood

Log-rank test (*p* = 0.0198)

According to subgroup analysis (see Table 3), even the cumulative proportion of HBV clearance was higher in heterozygous carriers than in wild-type controls among patients taking ETV, the difference was significant only at 12 weeks (OR, 2.33; 95% CI, 1.45–3.75; *p* < 0.001). However, the cumulative proportion of HBV clearance did not show a significant difference at 12, 24, 48, or 96 weeks between heterozygous carriers and wild-type controls among patients using TDF.

**FIGURE 4:**
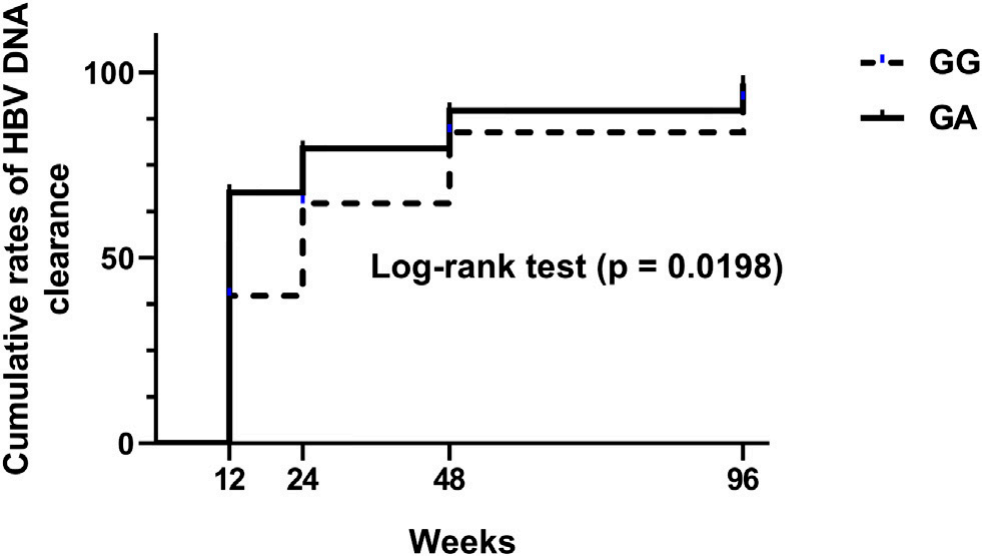
A Kaplan-Meier curve was plotted to assess the cumulative rate of HBV clearance (<20 IU/ml) at each time point, and the difference between the groups was assessed by a log-rank test.

Furthermore, a Kaplan-Meier curve (see Figure 4) was subsequently plotted according to Table 3 to assess the cumulative rate of HBV clearance at each time point, and then the difference between the groups was assessed by a log-rank test. The Kaplan–Meier analysis of the cumulative rate of HBV clearance showed that patients with the NTCP p.Ser267Phe variant exhibited faster HBV clearance than wild-type controls. The log-rank test results were significant (*p* = 0.0198).

In conclusion, CHB patients carrying the NTCP p.Ser267Phe variant had a higher HBV clearance rate than wild-type controls at 12 weeks, especially among those using ETV. Heterozygous CHB patients achieved HBV clearance significantly faster than wild-type controls.

Association between the NTCP p.Ser267Phe variant and HBV DNA decline in CHB patients on first-line NAs treatment

**FIGURE 5:**
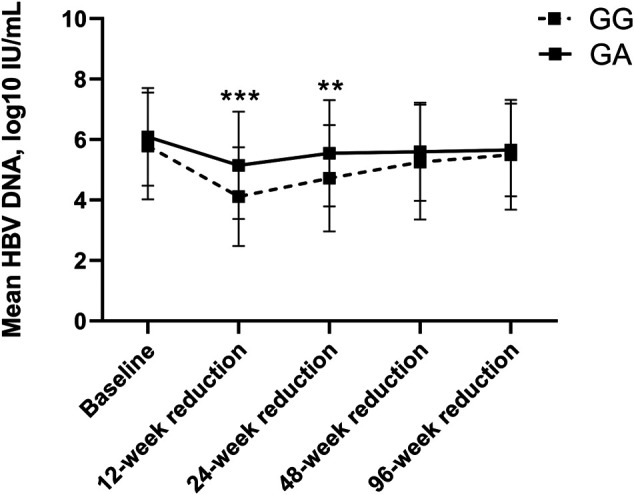
HBV DNA Reduction compared to baseline between wild-type control and heterozygote cases among CHB patients using first-line NAs.

To further assess the HBV DNA decline at each time point compared to baseline, the HBV DNA reduction at each time point was also examined and compared between groups (see Table 4 and Figure 5). According to Table 4 and Figure 5, HBV DNA declined significantly more at 12 and 24 weeks, with 5.15 ± 1.78 log10 IU/ml and 5.54 ± 1.76 log10 IU/ml, in heterozygous carriers, respectively, than in wild-type controls, with 4.11 ± 1.64 IU/ml (*p* < 0.001) and 4.72 ± 1.76 IU/ml (*p* = 0.006), respectively. Nevertheless, though the differences were not significant (*p* > 0.05), HBV DNA declined more in heterozygous carriers than in wild-type patients at the other time points, including 48 and 96 weeks.

**TABLE 4:** §HBV DNA Reduction between wild-type control and heterozygote cases.

	All subjects	GG	GA	*p* ^*#*^
**Baseline HBV DNA (68 pairs)**	**5.94** ± **1.69**	5.79 ± 1.76	6.09 ± 1.62	0.220
ETV (55 pairs)	5.88 ± 1.64	5.77 ± 1.63	5.98 ± 1.64	0.425
TDF (13 pairs)	6.16 ± 1.92	5.84 ± 2.32	6.52 ± 1.48	0.306
**12 weeks HBV DNA reduction (68 pairs)**	**4.63** ± **1.78**	4.11 ± 1.64	5.15 ± 1.78	**<0.001**
ETV (55 pairs)	4.62 ± 1.8	4.05 ± 1.64	5.18 ± 1.79	**<0.001**
TDF (13 pairs)	4.66 ± 1.76	4.34 ± 1.71	5.02 ± 1.8	0.374
**24 weeks HBV DNA reduction (68 pairs)**	**5.13** ± **1.81**	4.72 ± 1.76	5.54 ± 1.76	**0.006**
ETV (55 pairs)	5.19 ± 1.7	4.86 ± 1.62	5.52 ± 1.74	**0.036**
TDF (13 pairs)	4.87 ± 2.2	4.13 ± 2.26	5.62 ± 1.94	0.079
**48 weeks HBV DNA reduction (50 pairs)**	**5.48** ± **1.73**	5.25 ± 1.9	5.6 ± 1.63	0.314
ETV (38 pairs)	5.4 ± 1.69	5.23 ± 1.79	5.4 ± 1.66	0.663
TDF (12 pairs)	5.74 ± 1.9	5.32 ± 2.3	6.22 ± 1.42	0.245
**96 weeks HBV DNA reduction (43 pairs)**	**5.6** ± **1.87**	5.49 ± 1.82	5.65 ± 1.54	0.626
ETV (38 pairs)	5.6 ± 1.72	5.47 ± 1.75	5.68 ± 1.53	0.561
TDF (5 pairs)	5.57 ± 2.54	5.68 ± 2.55	5.47 ± 1.74	0.80

#*p* - value between GG and GA group. Significant p-value in bold (threshold p < 0.05).

^§^HBV DNA reduction defined as the HBV DNA load at each time point compared to baseline

**FIGURE 6:**
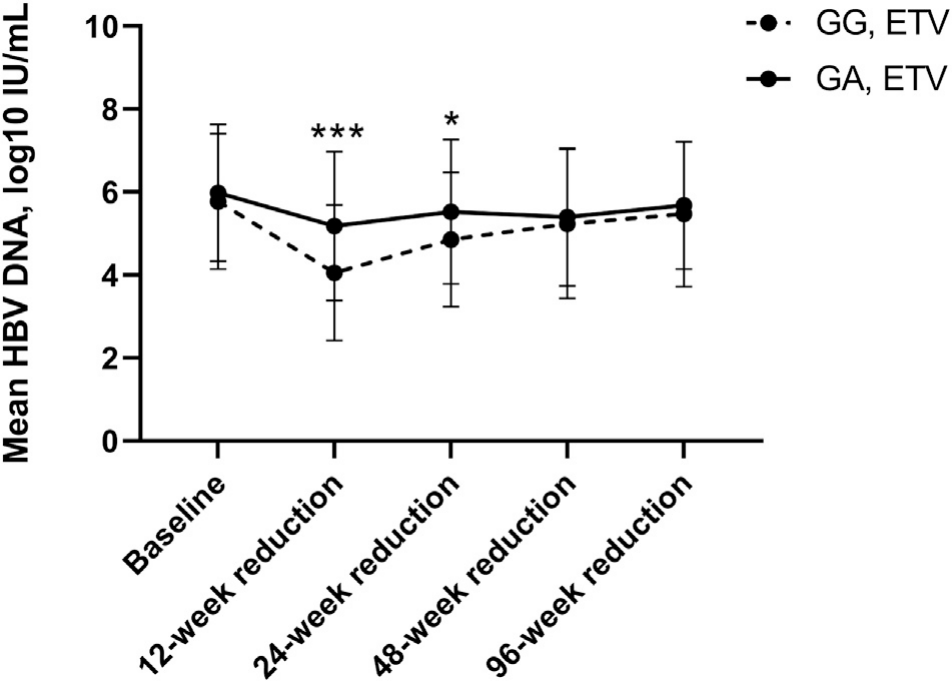
HBA DNA Reduction compared to baseline between wild-type control and heterozygote cases using ETV.

Similarly, in subgroup analysis for ETV (see Table 4 and Figure 6), HBV DNA declined significantly more at 12 and 24 weeks, with 5.18 ± 1.79 log10 IU/ml and 5.52 ± 1.74 log10 IU/ml, in heterozygous carriers, respectively, than in wild-type controls, with 4.05 ± 1.64 IU/ml (*p* < 0.001) and 4.86 ± 1.62 IU/ml (*p* = 0.036), respectively. Likewise, though the differences were not significant (*p* > 0.05), HBV DNA declined more in heterozygous carriers than in wild-type patients at other time points, including 48 and 96 weeks.

**FIGURE 7:**
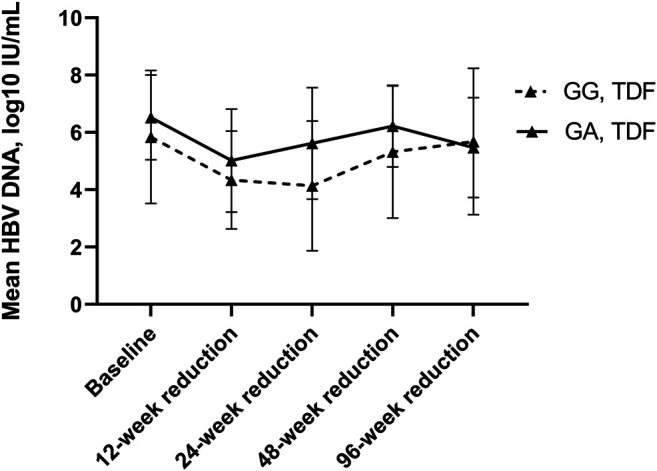
HBA DNA Reduction compared to baseline between wild-type control and heterozygote cases using TDF. Between group differences were not significant.

However, the HBV DNA decline in patients using TDF was not the same (see Table 4 and Figure 7). Though the differences were not significant (*p* > 0.05), heterozygous carriers using TDF exhibited a lower HBV DNA reduction at 12 weeks but a higher HBV DNA reduction at 24, 48 and 96 weeks than wild-type controls.

To summarize, we found the NTCP p.Ser267Phe variant carriers, especially those using ETV, had a greater HBV DNA decline at 12 and 24 weeks than wild-type controls.

## Discussion

Previously, our studies indicated that the NTCP p.Ser267Phe variant acts as a protective factor against HBV-related disease progression among CHB patients, in accordance with several other studies(Peng et al., 2015; Lee et al., 2017; An et al., 2018; Wu et al., 2018; Binh et al., 2019; Yang et al., 2019). However, the mechanism through which the mutation exerts a protective effect remained unclear. This study used propensity score matching to construct a comparable cohort and confirmed that the NTCP p.Ser267Phe variant carriers have a faster virological response than wild-type controls in HBV suppression upon treatment with NAs. Since many CHB patients receive oral antiviral treatment, this may be one of the reasons why the NTCP S267F variant protects CHB patients from aggressive disease progression.

Most interestingly, although all included NTCP S267F variant carriers were heterozygous and not homozygous in this study, none of them were primary nonresponders to NAs, and their HBV DNA levels reduced significantly faster at 12 and 24 weeks than the wild-type controls, suggesting that the mutation could exert a powerful anti-HBV effect among CHB patients during NAs treatment. However, the additive effect of the NTCP S267F variant did not occur in long-term treatment. First, we believe that on the hepatocyte surface of heterozygous carriers, only approximately half of the NTCP undergoes structural changes associated with the NTCP p.Ser267Phe variant, according to the central dogma of molecular biology. NAs prevent HBV replication and virion productivity, while relatively high numbers of new viruses/virions could infect hepatocytes through the normal NTCP of heterozygous carriers, causing a faster HBV DNA decline initially. However, as time passes (48 and 96 weeks after NAs treatment), with HBV replication eventually suppressed by NAs, new HBV virus/virions continue to diminish. At approximately week 48 or 96, of the presence or absence abnormal NTCP does not play an important role any longer. Second, HBV may evolve sophisticated means to evade or directly counteract the entry restriction factor NTCP and replication inhibitor NAs as a consequence of the continuous interaction with wild-type or mutant NTCP and NAs (Kluge et al., 2015; Jacquet et al., 2019; Takeuchi et al., 2019). Using phylogenetic analyses, Jacquet et al., (2019) even found that NTCP has been under recurrent positive selection during primate evolution, suggesting that one hallmark of pathogenic virus-host relationships is the reciprocal evolution of host receptors and viral envelope proteins as a result of their antagonistic interaction over time. Thus, with the long-term NTCP-HBV interaction and NAs-HBV interaction, some HBV may undergo adaptive changes (Yang et al., 2019), leading to drug resistance. Further research on the HBV sequence carried by heterozygous carriers with HBV recurrence is required.

Overall, our study demonstrated that NTCP S267F variant carriers exhibit faster anti-HBV effects than wild-type patients on first-line NAs treatment. Subgroup analysis showed that the significant effects were observed among patients using ETV but not TDF. For the inconsistent results between ETV and TDF, two explanations can be offered. First, we think that TDF has a temporary advantage of short-term efficacy because ETV has been on the market since 2006, which is eight years earlier than TDF in China. Compared to TDF, which has not yet been reported to confer resistance (Cathcart et al., 2018), ETV has several reported resistance-conferring mutations (Tenney et al., 2009), such as rtM204V/I/S ,rtL80I/V, rtV173L, and/or rtL180M, some of which may lead to steric hindrance for the second-line NAs lamivudine (Qiu et al., 2014). As it is attached to antibiotics, while the TDF-resistant HBV strain is rarely spread, we could assume that the efficacy of TDF is now so high that the NTCP p.Ser267Phe heterozygous variant has little ability to exert its addictive effect against HBV. Second, this may have been due to the relatively small sample size of CHB patients using TDF in our cohort. For example, no heterozygous carrier in the TDF treatment was a primary nonresponder, while among wild-type controls, the rate was as high as 30.77%. Since the trend is mostly in accordance with that of ETV (except for the virological breakthrough and reactivation of hepatitis B), we still think it necessary to perform this study. Nevertheless, these controversial inconsistencies need to be further investigated with the increasingly widely used TDF in China.

Oral nucleos(t)ide analog therapy is the main form of anti-HBV treatment worldwide. First-line NAs achieve high virological suppression in a high proportion of patients (more than 95% of patients achieve undetectable serum HBV DNA), with favorable safety and tolerability profiles and an extremely low incidence of virological breakthrough. However, drug-resistant variants may eventually emerge under antiviral pressure using nucleos(t)ide analogs (Seto et al., 2018). Once this happens, patients may continue with an old strategy, increase the dose or add other NA, but their effect and safety remain controversial (Chang et al., 2005). The use of drugs able to address different steps of the HBV life cycle is expected to hinder the dissemination of viruses with or without drug-resistant variants. Based on previous and present studies, the rs2296651 variant in SLC10A1 impairs binding between HBV and NTCP and exerts an additive anti-HBV effect on the host during NAs treatment. Therefore, HBV entry inhibition used in combination with NAs represents such an approach.

These result confirms that the NTCP p.Ser267Phe variant exerts additive antiviral effects among CHB patients on first-line NAs treatment, suggesting that the NTCP p.Ser267Phe variant exerts a protective effect on CHB patients partly by enhancing the antiviral effect in combination with NAs. Since back to 2013, Tassilo et al. (Volz et al., 2013) confirmed a better HBV suppression on humanized mice with sequential treatment of NTCP blocker myrcludex B and ETV. This presents the possibility of combining first-line NAs and the developing NTCP blocker for a better anti-HBV effect.

## Data Availability

The authors acknowledge that the data presented in this study must be deposited and made publicly available in an acceptable repository, prior to publication. Frontiers cannot accept a article that does not adhere to our open data policies.

## Abbreviations

HBV, Hepatitis B virus; NAs, Nucleos(t)ide analogs; NTCP, Sodium taurocholate co-transporting polypeptide; CHB, Chronic hepatitis B; ETV, Eanticavir; TDF, Tenofovir Disoproxil Fumarate; TAF, Tenofovir Alafenamide; SNP, Single nucleotide polymorphism; MAF, Minor-Allele Frequency; HBsAg, Hepatitis B surface antigen; PSM, Propensity Score Matching; OR, Odds ratios; 95% CI, 95% confidence intervals.
