# Supplementary material for: The NTCP p.Ser267Phe Variant Is Associated With a Faster Anti-HBV Effect on First-Line Nucleos(t)ide Analog Treatment
Source: Front Pharmacol. 2021 Feb 24;12:616858. doi: 10.3389/fphar.2021.616858 (PMC7943921; doi:10.3389/fphar.2021.616858)
Supplement: Supplementary file 1 [file datasheet1.pdf]

Supplementary materials

Table S1. The primers information used for the amplification of NTCP SNPs

**Method**	**SNP ID**	**Parameter**	**Strand(5′ →3′)**
Sanger Sequencing	rs2296651	Forward	CCATCTGCTGCGAAA CTC
		Reverse	GGGCTACCTGGTTCTT AGTGA
SnapShot	rs2296651	Forward	TTGTTGGCAGGCTCAGGTCTAA
		Reverse	CTTTGGTAGCAGCACTGGGACA

Table S2. The criteia of diagnosis and definitions of antiviral outcomes in our cohort

	Definitions	Reference
**Diagnosis**		
Chronic Hepatitis B, CHB	Seropositivity of hepatitis B surface antigen (HBsAg) over 6 months, without the presence of cirrhosis, hepatocyte carcinoma, and liver failure	Chinese Society Of Infectious Diseases, C. M. A., Chinese Society Of Hepatology, C. M. A., [The guidelines of prevention and treatment for chronic hepatitis B (2019 version)]. Zhonghua Gan Zang Bing Za Zhi 2019, 27, 938-961.
Cirrhosis	Evidence of decompensation such as image diagnosis of splenomegaly, portal hypertension ascites, hypoproteinemia (<36 g/L), or thrombocytopenia (<1 × 10^9/L)	Angeli, P., Bernardi, M., Villanueva, C., Francoz, C., et al., EASL Clinical Practice Guidelines for the management of patients with decompensated cirrhosis. J HEPATOL 2018, 69, 406-460.
Acute-on-chronic Liver Failure (ACLF)	Acute hepatic insult manifesting as jaundice (serum bilirubin≥5 mg/dL (85 mmol/L) and coagulopathy (INR≥1.5 or prothrombin activity < 40%)	Sarin, S. K., Choudhury, A., Sharma, M. K., Maiwall, R., et al., Acute-on-chronic liver failure: consensus recommendations of the Asian Pacific association for the study of the liver (APASL): an update. HEPATOL INT 2019, 13, 353-390.
Hepatocellular carcinoma	Diagnosed based on confirmed histopathological evidence by two independent pathologists, or at least two imaging examinations (hepatic ultrasound together with CT or MRI) with elevated serum α-fetoprotein level	[Guidelines for diagnosis and treatment of primary liver cancer in China (2019 edition)]. Zhonghua Gan Zang Bing Za Zhi 2020, 28, 112-128.
